# Retrieval of an Unusual Foreign Body

**DOI:** 10.7759/cureus.6110

**Published:** 2019-11-09

**Authors:** Paul D O'Connor, John Sciarra

**Affiliations:** 1 Anesthesiology, Larkin Community Hospital, South Miami, USA; 2 Anesthesiology, Larkin Community Hospital, South Miami, USA

**Keywords:** foreign body, cell phone, endoscopy, surgical management foreign body, community hospital, incarcerated population, inmates, sedation, outside of the or anesthesia

## Abstract

Foreign body (FB) ingestion represents a common presenting complaint of the incarcerated patient population treated at Larkin Community Hospital (LCH). These patients find an array of different objects to ingest, and some of these objects represent a significant cause of morbidity and mortality. Batteries, specifically, are a FB that may cause significant injuries if ingested, and thus urgent attention is required. The effects of swallowing small batteries are well documented in the literature. This is not the case for more complex electronic devices that contain a battery, such as a cell phone. One such example is described in a case where a 44-year-old male inmate ingested a small cell phone 12 days prior to arrival at LCH. This patient presented with minimal signs or symptoms on physical exam. The phone was removed by endoscopy under monitored sedation by the anesthesia and gastroenterology teams with surgery on standby. This case demonstrates the need for removal before the patient becomes symptomatic, as well as the interdisciplinary co-operation between general surgery and gastroenterology required to retrieve complicated battery-containing FBs, such as a phone, from the gastrointestinal tract following ingestion. This case also demonstrates that a complex object such as a phone may remain in the stomach for an extended time without being digested enough to cause severe symptoms under the special circumstances seen in this case.

## Introduction

Foreign body (FB) ingestion is a common presenting complaint of the incarcerated population seeking healthcare [1]. Ingestion of batteries is an important subtype of FB ingestion, and requires special considerations by the medical and surgical teams while treating such events. The primary focus of this case study is to highlight a rare type of FB ingestion, and discuss the importance of urgent multidisciplinary action to prevent significant morbidity and mortality related to caustic injury of the gastrointestinal (GI) tract. This particular case report describes one such event in which an uncommon type of battery ingestion that had a delayed presentation after the ingestion than typically described in the literature. Clinically, the patient had a good outcome in spite of the duration that the FB was exposed to the environment of the stomach and this case demonstrates how a complex FB that contains a battery may not behave as expected when compared to simple batteries.

## Case presentation

**Figure 1:**
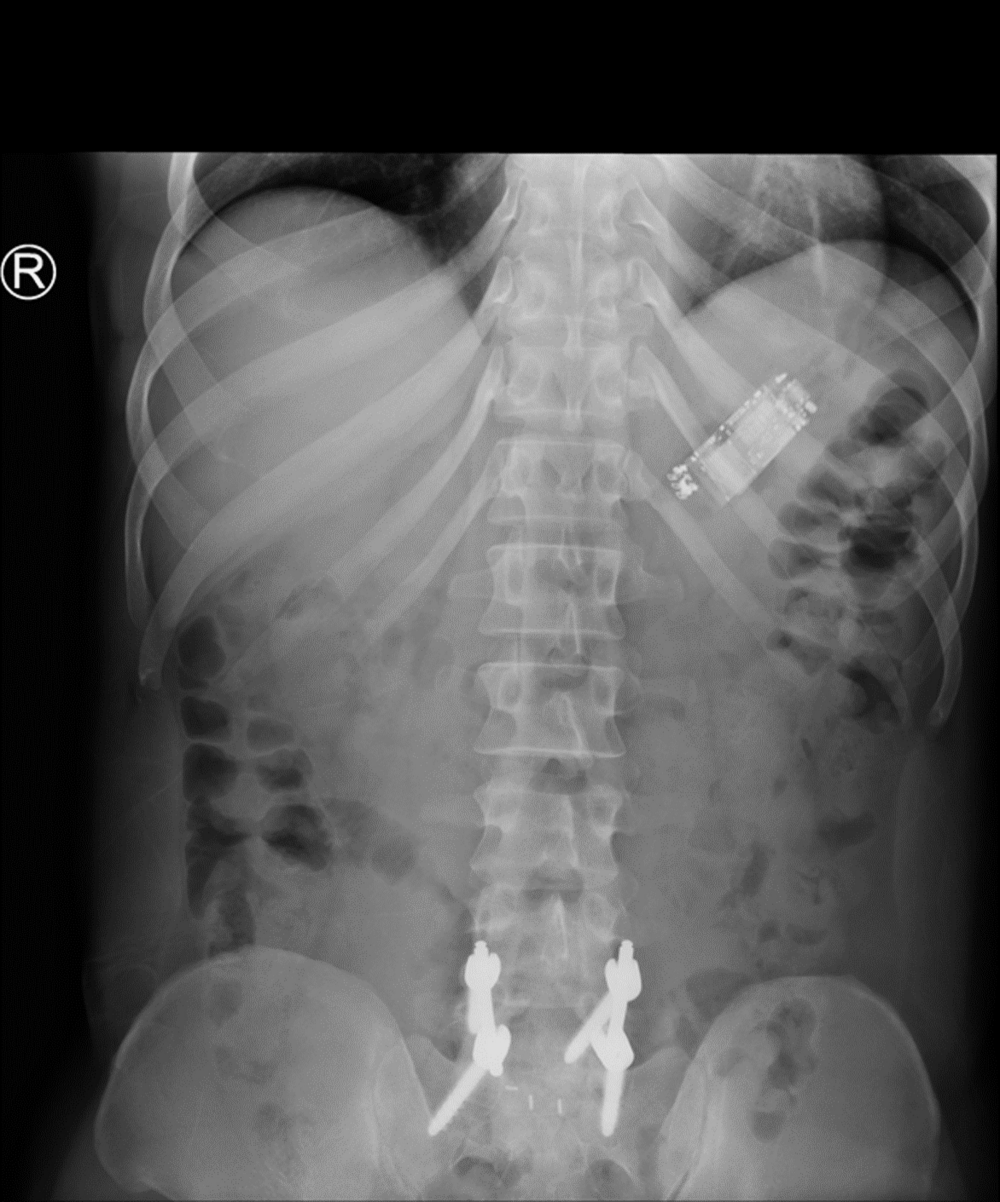
Initial KUB (abdominal plain X-ray). Revealed a radiopaque foreign body that appeared to be in the stomach. The patient was made NPO and general surgery was consulted. KUB, kidneys, ureters, and urinary bladder; NPO, nil per os.

**Figure 2:**
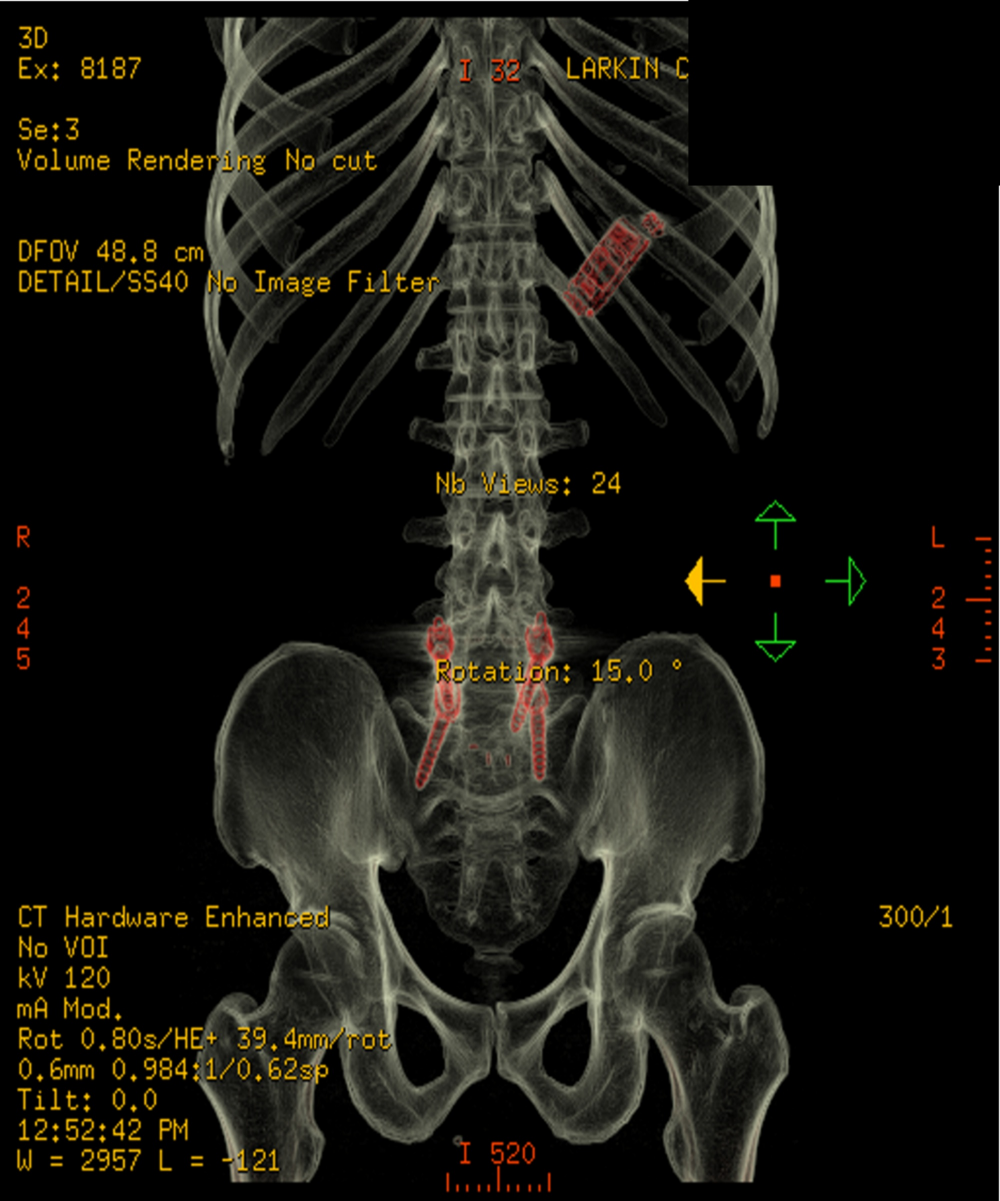
Hardware-enhanced CT. The foreign body is much more clearly delineated here using hardware-enhanced CT.

This was a 44-year-old male inmate with past medical history of sciatica and tubercolosis exposure on isoniazid who presented to Larkin Community Hospital (LCH) due to FB ingestion about 12 days prior. Upon interview the patient admitted to swallowing a cell phone. He complained of mild abdominal cramping and left lower quadrant abdominal pain. He denied nausea, vomiting, bloody stools, hematemesis, fever, or any other symptoms. Vital signs were within normal values at admission. Physical exam was only positive for mild tenderness to left lower quadrant without guarding, distension, or rebound. He was admitted and made nil per os (NPO) pending surgical evaluation. Initial plain film X-ray imaging showed a small, rectangular, radiopaque object in the patient’s stomach (Figure 1). Further CT imaging provided a much clearer image of the FB, in which the cell phone was clearly identifiable (Figure 2).

The patient was evaluated by the surgery team who recommended serial abdominal exams, bowel rest, pro re nata (PRN) pain control in addition to supportive therapy. Gastroenterology was consulted as well, and plans were made for endoscopic retrieval of the FB in the operating room (OR) with surgery on standby for robotic gastrotomy if esophagogastroduodenoscopy (EGD) retrieval was unsuccessful. Technical difficulties caused a delay in OR robotic availability so the case was performed in the endoscopy suite under monitored anesthesia care (MAC) sedation with propofol. The patient was monitored with pulse oximetry, electrocardiogram (EKG), capnography, and noninvasive blood pressure prior to sedation with propofol. Some 5 cc (50 mg) of 1% Lidocaine was sprayed into the patient’s throat prior to insertion of the endoscope. The anesthesia was carried out without complication. However, the patient required 1100 mg of propofol to remain sedated for the procedure which lasted about 1.5 h.

**Figure 3:**
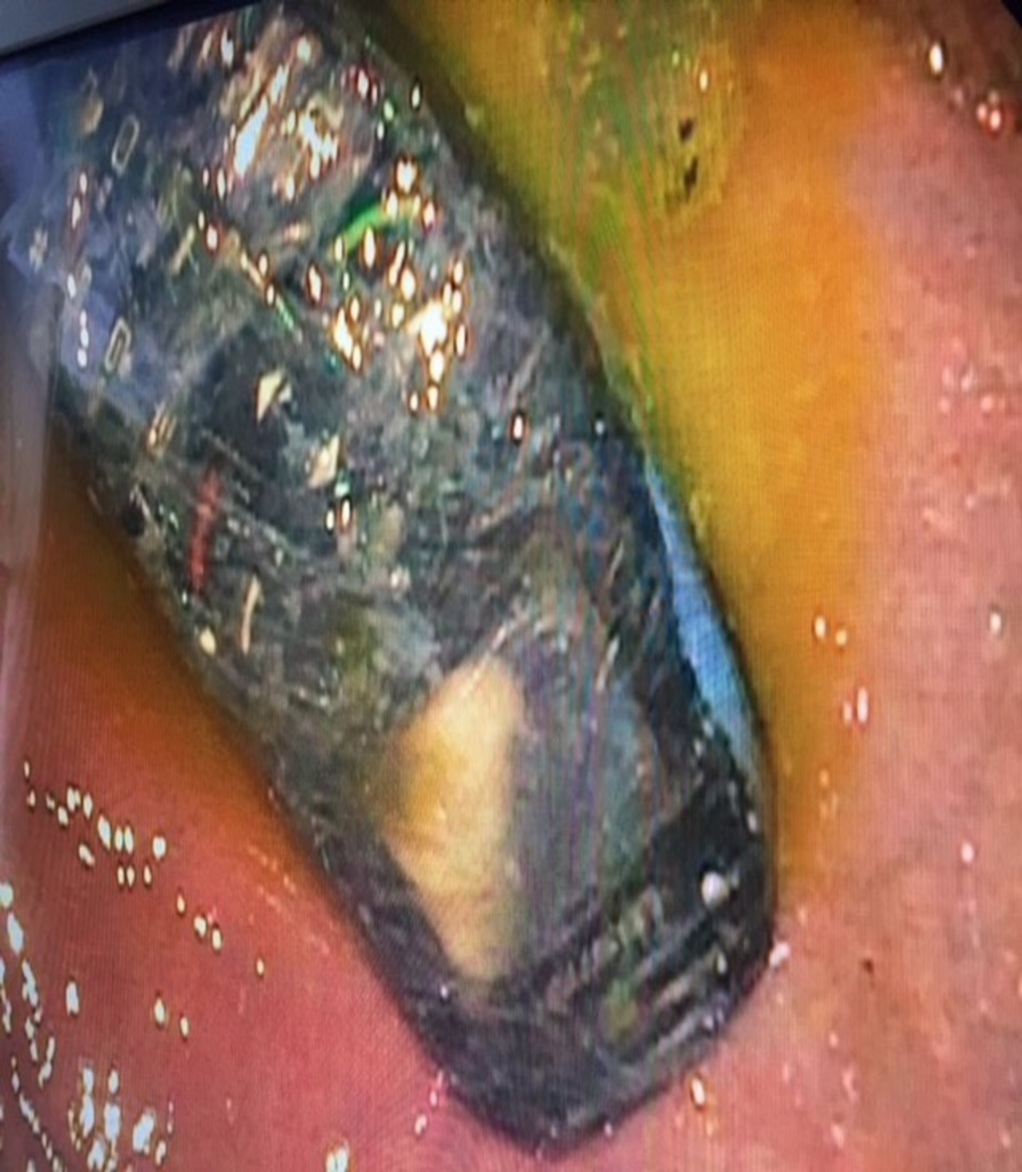
Endoscopic view. Note the phone was wrapped in a thin plastic covering which broke up during endoscopic manipulation.

The patient was placed in supine and left lateral recumbent position, and a plastic bite block was placed into his mouth prior to insertion of the endoscope. The FB retrieval itself was difficult due to the phone being wrapped in plastic, as shown in Figure 3.

**Figure 4:**
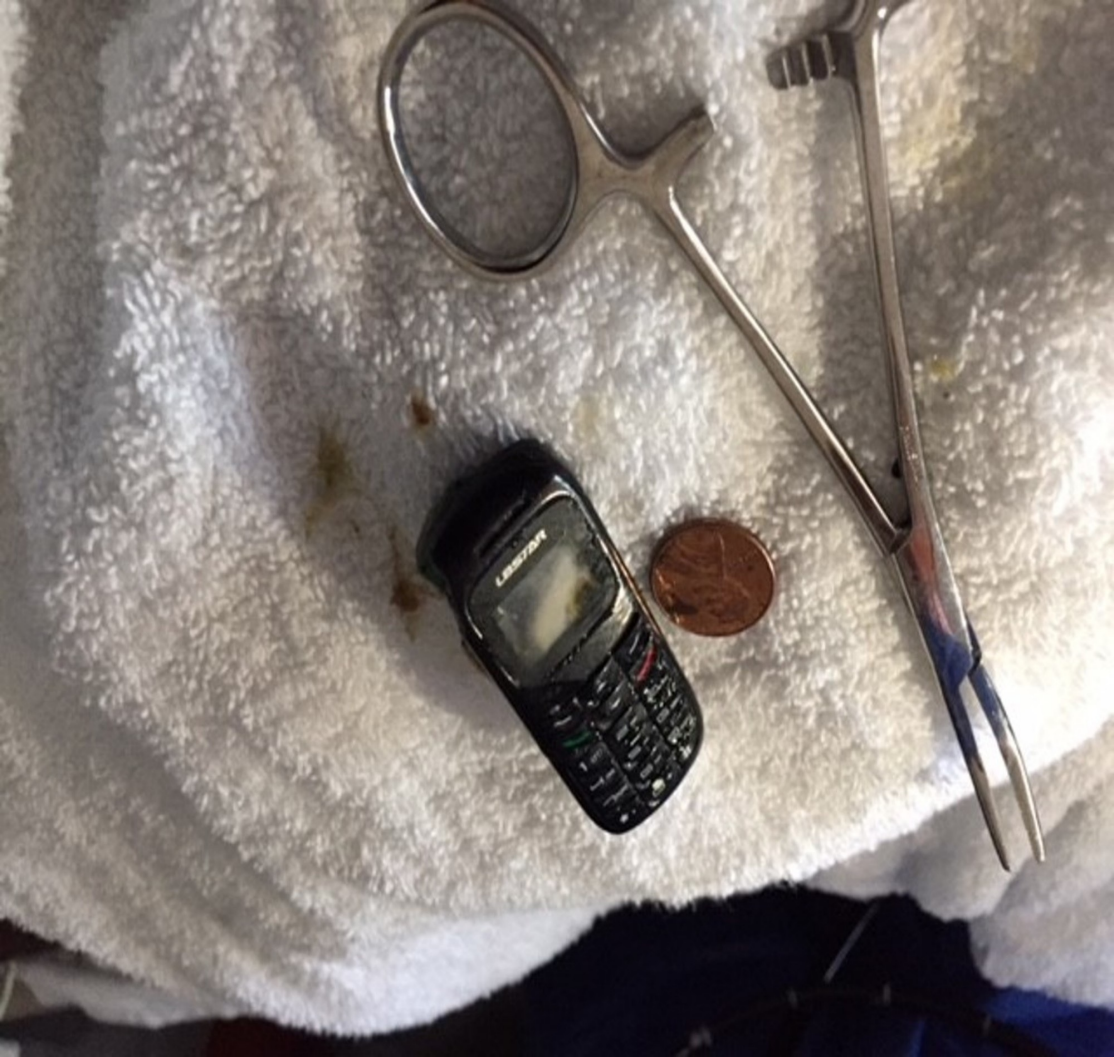
Retrieved phone. With penny and forceps for scale. The phone was about the size of a USB flash drive.

Retrieval was first attempted with the Trapezoid RX Boston Scientific 3 cm basket. The second attempt used a snare, which managed to grasp the phone and peel off the plastic covering. The third attempt using an alligator forceps was successful and removed the phone in a few large pieces due to breakup caused after the plastic covering was removed. Inspection of the FB upon removal revealed it to be a small cell phone approximately the size of a USB thumb drive. Figure 4 shows the gross FB outside of the patient with a penny for scale. Of note, the battery inside did not appear to show signs of content spillage. However, the phone internals were heavily damaged.

## Discussion

This case represents a rarely reported type of FB ingestion. The patient declined to provide motive for his actions but was presumed to be destroying evidence as per report from his guards. The literature describes both incarcerated and civilian adults with personality disorders who may exhibit similar attention seeking or potentially self-destructive behavior [1-2].

Battery ingestion specifically may cause severe injuries due to the GI tract as they are composed of metals such as lithium, zinc, mercury, and nickel. Different types of batteries may cause damage in different ways. For example, damage from ingestion of small lithium button batteries occurs due to formation of hydroxide ions at the anode, even if the battery casing remains intact [3]. Hydroxide ions then create a localized alkaline environment in the GI mucosa causing caustic injury and associated coagulative necrosis [4-5]. Other cylindrical batteries may cause spillage of their contents as the casing is destroyed by the digestive fluids of the GI tract, or by chewing upon ingestion. The alkaline contents cause damage to the GI mucosa in a similar fashion as the lithium disc batteries described above [4-5].

The American Society for Gastrointestinal Endoscopy (ASGE) guidelines provide a thorough overview of the management of battery ingestion. The guidelines state that observation of a stable patient, who specifically swallowed a cylindrical battery should be kept NPO and monitored by serial abdominal plain films to monitor progression of the battery and that endoscopic removal should be reserved for those who have failed to move after 48 h or the following situations: “Ingestion of cylindrical batteries with any signs of airway compromise, esophageal obstruction or perforation are an indication for emergency endoscopy, preferably with conscious sedation if appropriate [6].” “Emergency endoscopy is also indicated if there is any suspicion of damage to the battery from biting or chewing [6].” 

Small lithium disc batteries, however, pose a higher risk of causing damage by the mechanism described in the prior section. In these cases, it is recommended for endoscopic evaluation to take place within the first 12-48 h of ingestion. However endoscopy is not recommended between 5 and 15 days postingestion in these cases, as that is the period of time during which the tissue is at its weakest point after caustic injury [4-5]. In the case that the battery is lodged in the esophagus, retrieval should be attempted emergently, as caustic burns may appear within 2 h in this location [3]. These patients must be monitored for signs of complications such as esophageal rupture, mediastinitis, erosion into great vessels and potentially damage to the respiratory tract. These patients should also be closely monitored for later complications such as tracheoesophageal (TE) fistulas or strictures that may appear at 28+ days after removal of a lithium disc battery [3]. Of note, it is not recommended to induce vomiting, or to order lab checking for serum mercury or other metals [3].

Surgical standby and co-operation is important to manage foreign bodies when they are not amenable to endoscopic removal. The ASGE guidelines cite two recent studies that state approximately 12%-16% of foreign bodies will require surgical removal or surgical management of complications caused by the FB [6]. This case provides an example of such co-operation, as all teams were available and on standby should the need for surgical intervention arise. Another case report in the literature in which a cell phone was swallowed describes a situation in which the phone was unable to be removed and thus the patient required surgical removal. It is thus prudent to ensure the patient is consented for possible surgery prior to endoscopy as the situation may change in the endoscopy suite [7].

## Conclusions

Complex FBs such as a cell phone may contain other types of batteries than those described above. Small consumer electronics such as this may contain lithium polymer batteries. These batteries are generally larger and thus seemingly less likely to be ingested. Therefore, not much data currently exists in the literature about the sequela of lithium polymer battery pack ingestion. This case represents one such event, where a lithium polymer battery pack was ingested and exposed to the gastric environment for approximately 12 days without showing signs of leakage or caustic injury to the underlying mucosa. Failure to retrieve the phone in this patient may have had devastating results if the battery was exposed to his stomach acid. However, the plastic coating on the phone seems to have contributed to its durability in the gastric environment when compared to batteries more commonly ingested.
